# Comparing the Effectiveness of Different Dentinal Desensitizing Agents: *In Vitro* Study

**DOI:** 10.1155/2021/6652250

**Published:** 2021-02-12

**Authors:** Mohideen S. Farook, Okba Mahmoud, Maysara Adnan Ibrahim, Marwah Berkathullah

**Affiliations:** ^1^Faculty of Dentistry, University of Malaya, Kuala Lumpur, Malaysia; ^2^College of Dentistry, Ajman University, Ajman, UAE; ^3^College of Dental Medicine, University of Sharjah, UAE; ^4^Department of Restorative Dentistry, Faculty of Dentistry, Baqai Medical University, Karachi, Pakistan

## Abstract

**Objectives:**

To evaluate the *in vitro* effectiveness of desensitizing agents in reducing dentine permeability.

**Methods:**

The efficacy of desensitizing agents in reducing dentine permeability by occluding dentine tubules was evaluated using a fluid filtration device that conducts at 100 cmH_2_O (1.4 psi) pressure, and SEM/EDX analyses were evaluated and compared. Forty-two dentine discs (*n* = 42) of 1 ± 0.2 mm width were obtained from caries-free permanent human molars. Thirty dentine discs (*n* = 30) were randomly divided into 3 groups (*n* = 10): Group 1: 2.7% wt. monopotassium-monohydrogen oxalate (Mp-Mh oxalate), Group 2: RMGI XT VAR, and Group 3: LIQ SiO_2_. Dentine permeability was measured following treatment application after 10 minutes, storage in artificial saliva after 10 minutes and 7 days, and citric acid challenge for 3 minutes. Data were analysed with a repeated measures ANOVA test. Dentine discs (*n* = 12) were used for SEM/EDX analyses to acquire data on morphological changes on dentine surface and its mineral content after different stages of treatment.

**Results:**

Desensitizing agents' application on the demineralized dentine discs exhibited significant reduction of permeability compared to its maximum acid permeability values. Mp-Mh oxalate showed a significant reduction in dentine permeability (*p* < 0.05) when compared to RMGI XT VAR and LIQ SiO_2_. On SEM/EDX analysis, all the agents formed mineral precipitates that occluded the dentine tubules.

**Conclusions:**

2.7% wt. monopotassium-monohydrogen oxalate was significantly effective in reducing dentine permeability compared to RMGI XT VAR and LIQ SiO_2_.

## 1. Introduction

Dentine sensitivity is a common problem in an adult population with a prevalence ranging from 8 to 57% [1, 2]. The pain is mostly experienced by individuals who have permeable and exposed dentine due to tooth surface loss. Normally, the pain results from the stimulation of exposed dentine and its tubules via pulpal nerves by external stimuli such as chemical, thermal, tactile, mechanical, evaporative, or osmotic means which cannot be attributed to any form of dental defect or pathology [3, 4]. The patient usually represents with a complaint of short, sharp, and transient pain which is spontaneous when triggered by a stimulus followed by a deep dull pain. This pain is perhaps localized to few teeth or could be generalized affecting multiple surfaces of the teeth [5]. Dentine sensitivity is the term used to characterize an unpleasant sensation developed in the dentine which previously responded as normal. However, “hypersensitive dentine” is the term used when the patient develops exaggerated unpleasant sensation compared to previous history of sensitivity [6].

The widely accepted theory for dentine sensitivity proposed by Brännström et al. [7] had explained that any thermal, tactile, chemical, osmotic, or evaporative stimuli applied on the exposed dentine stimulates the dentinal fluid movement. Such movement leads to the changes in intra pulpal pressure which in turn triggers the nerve receptors near the pulp causing sharp and well-localized tooth pain. Hence, a significant assumption of hydrodynamic theory is that the sensitive dentine is permeable [8]. Based on this theory, any reduction in dentinal fluid movement should decrease dentine permeability [9].

Different approaches are undertaken in the dental practices to treat dentinal sensitivity such as application of anti-inflammatory agents, nerve desensitizers, dentine sealers, ion/salts, protein precipitants, laser, soft tissue grafting, and restorative agents [10]. The vast majority of the treatment is based on the mechanism to occlude the dentinal tubules. Regardless of the different treatment agents, dentine sensitivity often reoccurs due to mechanical challenges from excessive tooth brushing, during periodontal therapy such as scaling and root surface debridement, chemical erosion, or mechanical dislodgement of the coated material leading to short-term desensitizing effects [11, 12]. It is essential that the dental products could chemically interact with the dental tissues to form a stable structure that could resist the fluid movements within the dentinal tubules.

The desensitizing agents such as resin-modified glass ionomer (Clinpro XT Varnish) and liquid SiO_2_ complex (Dentcoat DSP) were introduced in the market between 5 and 10 years' time to treat dentine sensitivity. Up to now, scant data in the literature report in vitro studies comparing LIQ SiO_2_ efficacy on dentine hypersensitivity with other dentine permeability treatments.

Therefore, the aim of the study was to evaluate the effectiveness of the desensitizing agents under various conditions that simulates the oral environment in the reduction of dentine permeability and tubular occlusion.

## 2. Materials and Methods

Clinpro XT Varnish is a resin-modified and light-cured glass ionomer varnish which has two main components, methacrylate-modified polyalkenoic acid and hydroxyethyl methacrylate (HEMA) in addition to fluoroaluminosilicate which helps to bond the materials to the dentine both chemically and mechanically [13]. Besides that, its high fluoride release has the potential to protect the teeth from root caries.

Dentcoat DSP is a compound of the liquid SiO_2_ (silicon dioxide) complex. It acts by forming a biomimetic protective coating that can effectively protect enamel from acid challenge. The coating is biorepulsive in nature and prevents the adherence of plaque and microorganisms.

Its mechanism of action in reducing sensitivity is by the formation of silica crystals on the exposed dentinal surface and tubules via the process of hydrolysis.

### 2.1. Preparation of Potassium Oxalate Solution

It was prepared by using 5% oxalic acid (pH 1.2) and titrated to pH 2.4 by adding potassium hydroxide (KOH) to form 2.7% wt. monopotassium-monohydrogen oxalate.

### 2.2. Preparation of Dentine Samples

Forty-two dentine discs (*n* = 42) were prepared from extracted sound human molars which were removed because of surgical indications in patients with recurrent pericoronitis and severe periodontal disease. The teeth were collected from oral surgery and a primary care unit in the University of Malaya dental hospital in a two-month period during 2018 and stored in 1% chloramine-T solution for no longer than 3 to 5 months following the extraction. The study design was approved by the Ethics Committee of the Faculty of Dentistry, University of Malaya (DF RD1507/0023(U)). The teeth were sectioned in a transverse plane using a diamond disc in a slow speed saw attached to water coolant (Micracut125 Metkon, University of Malaya). Dentine thickness of 1 ± 0.2 mm was obtained by removing the coronal portion of the sample, 1 mm below the occlusal pit, and the root segment was sectioned 2 mm below the cementoenamel junction. Necrotic tissues within the pulp chamber were extirpated using barbed broach, ensuring that its inner wall was not damaged. During the process of sample preparation, any critical damage to the samples was excluded from the study.

The dentine sample was glued using a cyanoacrylate-based adhesive (Ruichang Dei Adhesive Co., Ltd., China) to an acrylic Plexiglass (YSME Sdn Bhd, Malaysia) sized 1.5 cm × 1.5 cm × 0.5 cm. The center of the Plexiglass was drilled and fitted with 1.5 cm length of 18-gauge stainless steel tube which would aid in the passage of fluid from the device into the pulp chamber. Flowable composite (3M ESPE FiltekZ350XT, USA) was placed around the radicular portion of dentine samples to prevent any leakage during the measurement [14].

### 2.3. Fluid Filtration Device

**Figure 1:**
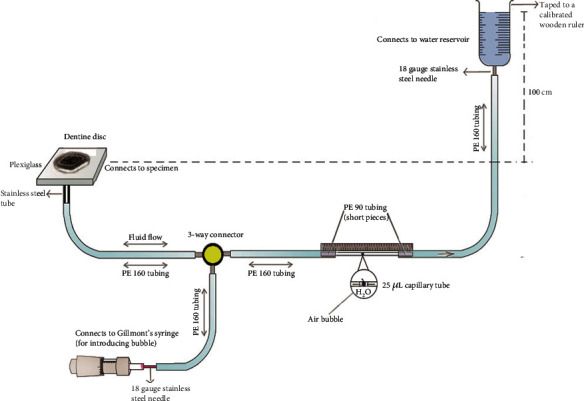
Experimental framework of “fluid filtration device.”

Dentine permeability (Lp) for the prepared dentine disc treated with different desensitizing agents was investigated using a fluid filtration device (Figure 1) working at 1.422 psi (100 cm H_2_O) pressure [15]. Polyethylene tubing (PE tube) of the filtration system was connected to the dentine disc via an 18-gauge stainless steel tube. A 25 *μ*m microcapillary tube (Microcaps, Fisher Scientific, Atlanta, GA, USA) in the system was attached on the right side to the water reservoir at the height of 100 cm from the dentine disc to create a pressure of 1.422 psi and the left side was linked to dentine specimen through a 3-way connector. Gillmont's syringe (Thermo Scientific, USA) which helps to introduce and control the air bubble inside the capillary tube was attached to a different outlet of the same connector via the PE tube.

### 2.4. Experimental Design

#### 2.4.1. Dentine Permeability Analysis for Treated Dentine

**Figure 2:**
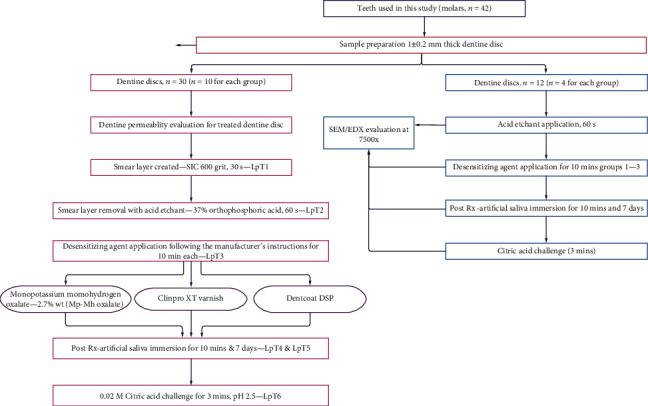
Flowchart of study design for analysing dentine permeability (LpT) and SEM/EDX of various dentine treatments.

Thirty dentine samples were randomly assigned for each group (*n* = 10).  Figure 2 shows the flow chart of the study design for investigating dentine permeability after various stages of treatment.

Minimum permeability value (LpT1) for the dentine disc was measured by creating a smear layer on the dentine surface using abrasive paper coated with 600 grit silicon carbide for 30 seconds [16]. Maximum permeability value (LpT2) was evaluated by treating the specimen with 37% orthophosphoric acid (3M7423 Scotchbond etchant) for 60 seconds [17].

Desensitizing agents were applied on the samples for 10 minutes based on the manufacturer instructions, and the dentine permeability was assessed (LpT3).  Table 1 shows the active ingredients of the agents. Subsequently, the dentine disc was stored in the artificial saliva at 37°C for 10 minutes and 7 days, and the permeability assessments were performed to determine the values for LpT4 and LpT5. The composition of artificial saliva (Alfatech Sdn Bhd, Malaysia) is CaCl_2_ 1.5 mmol/L, KCL 50 mmol/L, KH_2_PO_4_ 0.9 mmol/L, and Tris 20 mmol/L (19). Final evaluation (LpT6) was performed by subjecting dentine discs to 0.02 M citric acid solution (pH 2.5) for 3 minutes.

During the experiment, dentine permeability (Lp) was measured by the movement of a bubble within the capillary tube which was observed against a calibrated ruler incremented and measured in mm and converted into volume displacement as shown in the formula below. The bubble movement was measured for 4 minutes with three consecutive measurements after the system had been stabilized for the first minute. The dentine permeability for treated dentine (LpT) was calculated by dividing the fluid flow (*μ*L) by the surface area of dentine exposed (cm^−2^) and hydrostatic pressure (cmH_2_O/psi). The permeability of each dentine disc was expressed as percentage (LpT%) of the fluid flow across the acid-etched dentine disc of the same specimen. Therefore, each dentine disc acted as its own control.

### 2.5. SEM/EDX Analysis

Dentine discs (*n* = 12) were prepared similar to the samples prepared for dentine permeability evaluation. The dentine discs (*n* = 4) were randomly selected, prepared, and processed to analyse the morphological changes on the dentine surfaces and its mineral content following acid etching for 60 seconds, application of desensitizing agents for 10 minutes according to the manufacturer's instructions, storage in artificial saliva (10 minutes and 7 days), and citric acid challenge for 3 minutes. Each dentine disc was air-dried in a sun-dry cabinet at a constant room temperature 37°C and sputter coated with gold in a vacuum evaporator (Polaron Q150RS, Hi-Tech Instruments Sdn Bhd, Malaysia). The dentine morphology and its mineral content were analysed using a scanning electron microscope (SEM) (Quanta FEG 250, Crest Co., Holland) equipped with an energy X-ray dispersive spectrometer EDX (Oxford X-Max using Inca software, Crest Co., Holland). The machine was operated at the voltage of 10 kV, and the SEM images were captured at the magnification of 7500x. All the samples were analysed in the horizontal section except for the dentine specimens treated with desensitizing agents, which were viewed both in the horizontal and longitudinal sections by fracturing the specimens.  Figure 2 shows the study design for the SEM/EDX analysis.

### 2.6. Statistical Analysis

Statistical analysis was performed using SPSS version 25 for Windows. The permeability data (LpT3, LpT4, LpT5, and LpT6) were transformed into percentages of the original LpT2 (maximum permeability) values. Means and standard deviations of LpT values were calculated for each group. Homogeneity of variance was assessed by using Levene's test (*p* > 0.05). One-way ANOVA was used to compare the significant differences between the groups (*p* < 0.05). Repeated measures ANOVA was used to analyse within the group differences (*p* < 0.05). Post hoc multiple comparisons were conducted using Tukey and Dunnett's T3 test. All *p* values were set at 0.05.

## 3. Results

### 3.1. Dentine Permeability Analysis for Treated Dentine

All the desensitizing agents showed reduction in the permeability values of the dentine.  Table 2 shows the dentine permeability values for each treatment stages. Acid etch treatment on the tubular surface of the dentine had increased the permeability values to its maximum level equivalent to 100% (arbitrary value). Therefore, the values for the dentine permeability were expressed as percentages of its maximum permeability obtained in LpT2 treatment. Each acid-etched dentine represented its own control of that specimen. All the materials showed decrease in the dentine permeability after its application on acid-etched dentine (LpT2). However, the LIQ SiO_2_ showed only 15% of reduction whilst the other agents such as Mp-Mh oxalate and RMGI XT VAR showed 92% and 85%, respectively. There is a significant difference in the reduction in the permeability between the groups at this point of treatment (*p* < 0.05). Submersion in artificial saliva for 10 minutes increases the dentine permeability for all the groups except for LIQ SiO_2_. Seven days of submersion in artificial saliva showed decrease in dentine permeability for Mp-Mh oxalate and RMGI XT VAR by 1% and 2%, respectively, whereas LIQ SiO_2_ showed 22% increase in the dentine permeability. Eventually, citric acid challenge (LpT6) for all the specimens showed increase in the permeability of the dentine. Amongst all the groups, Mp-Mh oxalate showed a significant effect on the dentine permeability reduction for the LpT3, LpT4, LpT5, and LpT6 stages. However, there is no significant difference in the permeability values within the group at different stages of treatment except between the LpT2 and LpT3 stages.

### 3.2. SEM/EDX Analysis

**Figure 3:**
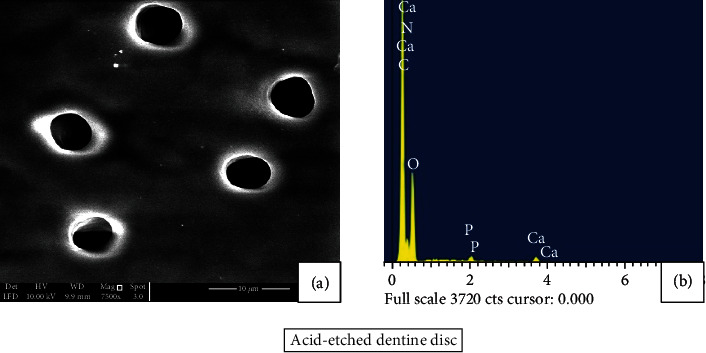
(a) SEM image (7500x) and (b) EDX analysis. Dentine disc representing the patency of dentine tubules following the acid etching EDX analysis revealed high and low peaks of Ca and P, respectively.

The formation of mineral precipitates and its effect on open dentinal tubules were further analysed using scanning electron microscopy. Composition of the mineral precipitates was investigated using an element-sensitive detector (EDX). The dentine disc was demineralized using 37% orthophosphoric acid that resulted in wash out of the smear layer and exhibited entirely patent dentine tubules (Figure 3(a)). EDX spectra show high and low peaks of Ca and P, respectively (Figure 3(b)).

**Figure 4:**
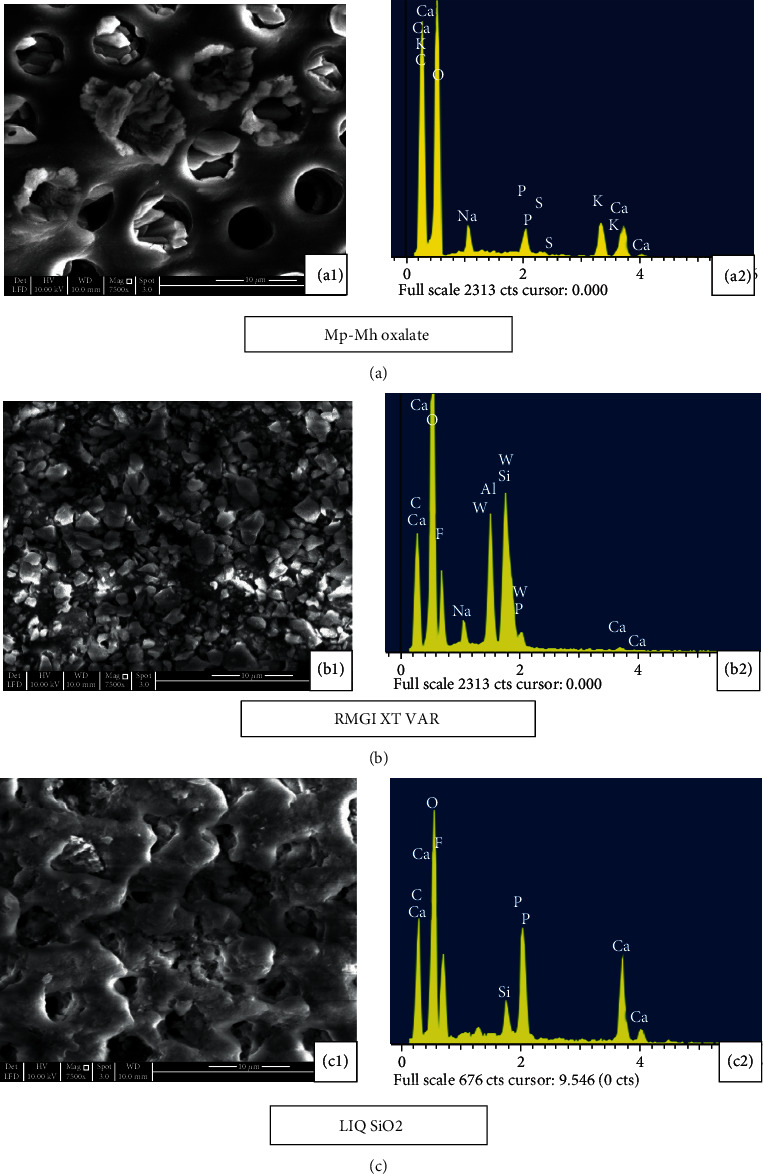
SEM images (7500x) and EDX analysis of the dentine disc following the application of desensitizing agent. (a1) The etched dentine disc treated with Mp-Mh oxalate exhibited dense snowflake-like calcium oxalate crystals occluding dentine tubules whilst few tubules appeared patent. (a2) EDX analysis revealed high peaks of Ca and K. (b1) The application of RMGI XT VAR exhibits crystals like a cobblestone pattern masking the entire dentine surface. (b2) EDX analysis revealed a high level of Ca, Al, and Si with a low level of Na and P. (c1) LIQ SiO_2_ exhibit occlusion of dentine tubules by amorphous deposits with few vacant tubules. (c2) EDX analysis revealed a high peak of Ca, P, and F with a moderate level of Si.

**Figure 5:**
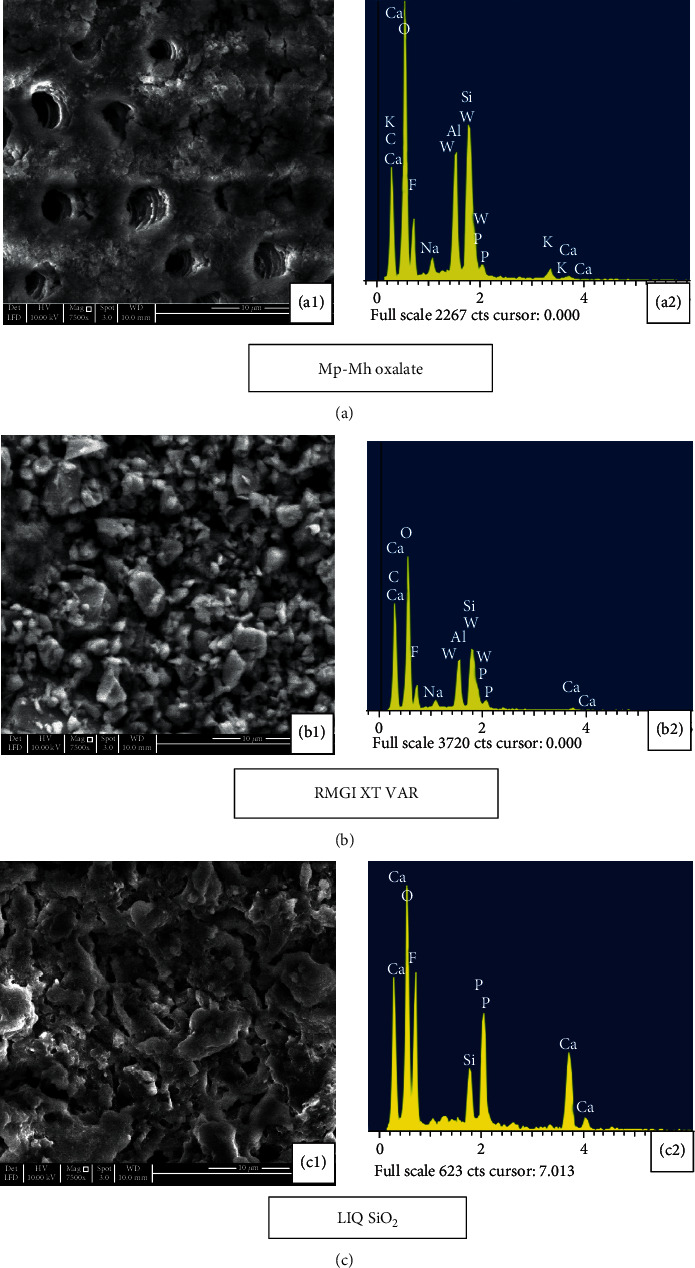
SEM images (7500x) and EDX analysis of treated dentine disc immersed in artificial saliva for 10 minutes. (a1) Mp-Mh oxalate shows oxalate precipitate blocking some tubules while others are patent. (a2) EDX spectra show a high peak of Ca and K along with other minerals such as Al, Si, and F. (b1) RMGI XT VAR shows the presence of crystal deposits on the dentine surface with few open tubules. (b2) EDX spectra show a moderate level of Ca, Al, and Si. (c1) LIQ SiO_2_ show Si precipitate masking the dentine surface with few open tubules. (c2) EDX spectra show a high level of Ca, P, and F with a moderate level of Si.

Application of the Mp-Mh oxalate on etched dentine disc resulted in the formation of dense snowflake-like calcium oxalate crystals occluding dentine tubules few microns deep within and outside the tubule walls whilst few tubules appeared patent (Figure 4(a1)). EDX analysis revealed the prevalent mineral content of Ca and K (Figure 4(a2)). Some oxalate precipitates within the dentinal tubules appeared to withstand the immersion in artificial saliva for 10 minutes and 7 days with some patent tubules seen on the dentine surface (Figures 5(a1) and 6(a1)). A high peak of Ca and K along with other minerals such as Al, Si, and F was evident (Figure 5(a2)).

**Figure 6:**
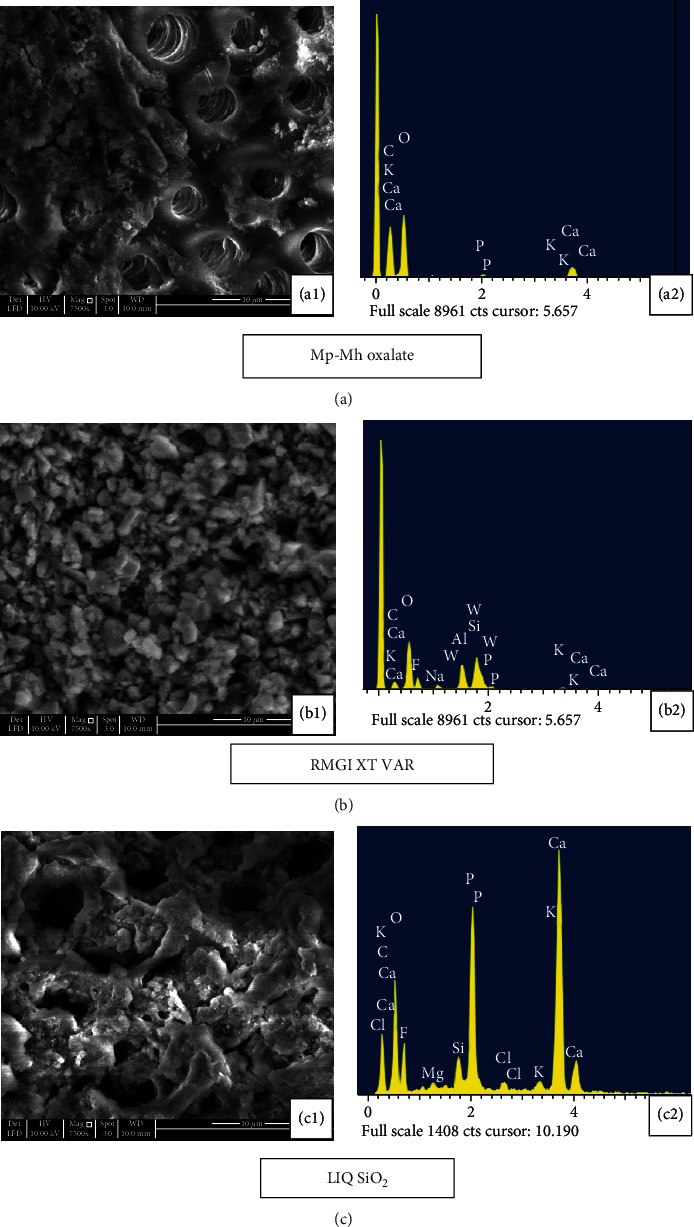
SEM images (7500x) and EDX analysis of treated dentine disc following the immersion in artificial saliva for 7 days. (a1) Mp-Mh oxalate shows the oxalate precipitates over few dentinal tubules with some patent tubules. (a2) EDX analysis revealed moderate peaks of Ca and K and a low level of P. (b1) RMGI XT VAR show the cobblestone pattern of crystals covering the entire dentinal surface with few open tubules. (b2) EDX analysis revealed a low level of Ca, Al, and Si. (c1) LIQ SiO_2_ show interconnected Si precipitate with few open tubules. (c2) EDX analysis revealed high levels of Ca, P, F, and K along with a low level of Si.

**Figure 7:**
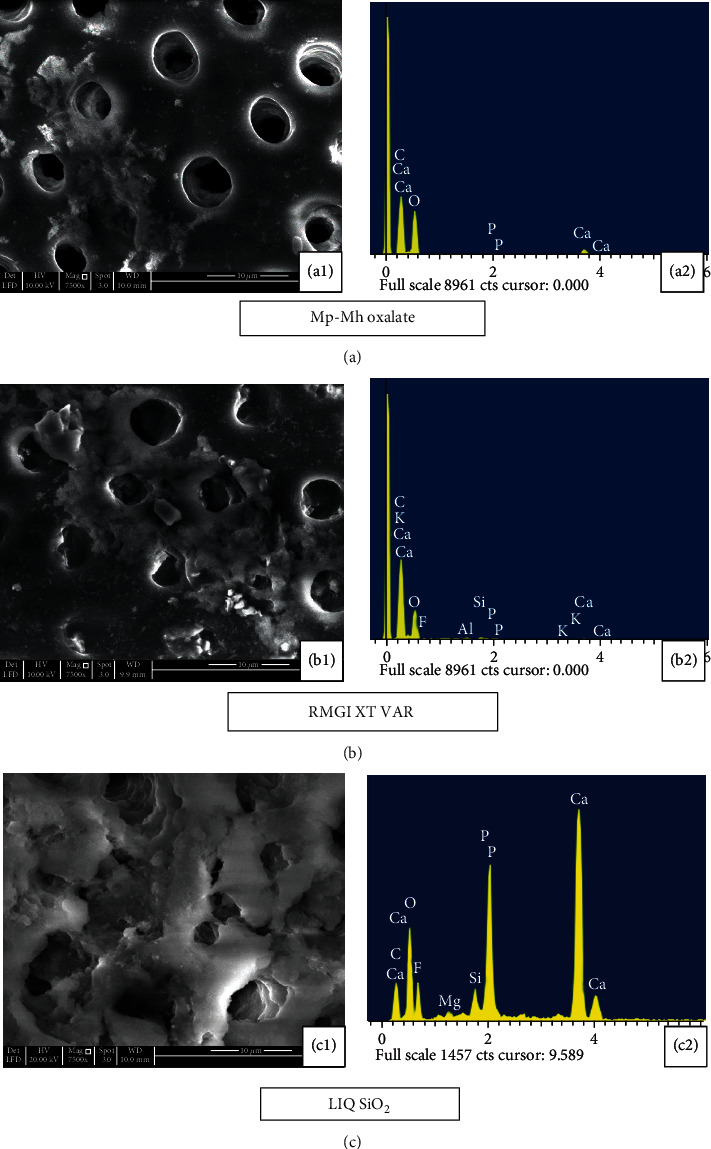
SEM images (7500x) and EDX analysis of treated dentine disc following citric acid challenge for 3 minutes. (a1) Mp-Mh oxalate resulted in dissolution of the tubular plugs, yet some crystal deposits could be seen within the tubule. (a2) EDX analysis revealed a moderate level of Ca and traces of P. (b1) RMGI XT VAR show loss of crystal structures on the dentine surfaces with few deposits within the tubules. (b2) EDX analysis revealed a moderate level of Ca and K with traces of F, Al, Si, and P. (c1) LIQ SiO_2_ show the change in the morphology of the deposits covering dentine surfaces exposing few tubules. (c2) EDX analysis revealed high peaks of Ca and P and a low peak of Mg, Si, and F.

**Figure 8:**
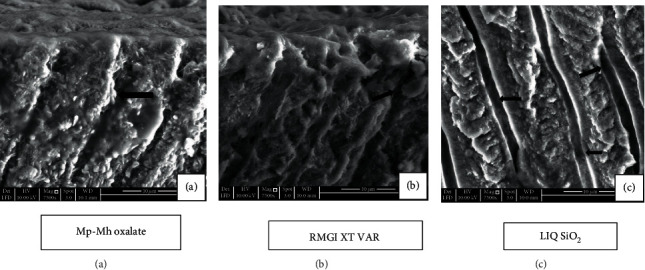
SEM image (7500x) of longitudinal section of etched dentine specimen treated with desensitizing agent for 10 minutes. (a) Mp-Mh oxalate exhibits the presence of oxalate crystals within the length of the tubules (arrowhead). (b) RMGI XT VAR shows some mineral deposits within the tubules (arrowhead). (c) The tubules appear to be vacant transversely along its length (arrowhead).

The minerals which were formed during the previous treatment had been reduced following storage in artificial saliva for 7 days (Figure 6(a2)). Apparently, more tubular openings and less mineral content are seen after the citric acid challenge (Figures 7(a1) and 7(a2)). Longitudinal section of the etched dentine specimen treated with Mp-Mh oxalate showed the presence of calcium oxalate crystals filling tubules along its length (Figure 8(a)).

Etched dentine discs treated with RMGI XT VAR resulted in the formation of a mineral precipitate with a cobblestone-like pattern which exhibited high peaks of Ca, F, Al, and Si (Figure 4(b2)) masking the entire surface of dentine and its tubules (Figure 4(b1)). Storage in artificial saliva for 10 minutes and 7 days revealed very few apparent patent tubules in SEM images (Figures 5(b1) and 6(b1)). However, in EDX spectra, this mineral content was reduced after the immersion in artificial saliva for 10 minutes and 7 days (Figures 5(b2) and 6(b2)). Subsequent acid challenge further lowered the minerals (Figure 7(b2)), and the dentinal tubules were exposed with the presence of some deposits within the tubules (Figure 7(b1)). Cross-section of the etched dentine samples treated with RMGI XT VAR demonstrated that the lateral walls of the dentine tubules below the superficial surface were being devoid of crystal particles except for some areas (Figure 8(b)).

The SEM image of LIQ SiO_2_ treatment reveals the presence of amorphous deposits of silica blocking the tubules and covering the dentine surfaces (Figure 4(c1)). The deposits show a high peak of Ca, P, and F with a moderate level of Si (Figure 4(c2)). Mineral content for this material was improved after being immersed in artificial saliva for 10 minutes and 7 days (Figures 5(c2) and 6(c2)). However, a few particles in between the precipitate got disintegrated and eventually exposed the tubules (Figures 5(c1) and 6(c1)). The mineral content was consistent even after citric acid challenge showing high peaks of Ca and P and low levels of F and Si with traces of Mg (Figure 7(c2)). The SEM image demonstrated the existence of the mineral precipitate on the occlusal surface of the tubule with a moderate number of tubules exposed (Figure 7(c1)). The cross-section of the treated dentine disc with LIQ SiO_2_ revealed the tubules being completely devoid of silica deposits below the surface (Figure 8(c)).

## 4. Discussion

This experiment employed the fluid-filtering system that was suggested by Pashley et al. [20, 18] which is an investigation tool to assess the quantitative changes within and outside the surface of the dentine tubules. It is a useful and potential method for the evaluation of the dentine permeability reduction by desensitizing agents as a result of dentine tubule occlusion.

As suggested by Taher et al. [21 [19]. This experiment used 100 cmH_2_O pressure since it produces considerably higher mean permeability values compared to the physiological pulpal pressure of 14 cmH_2_O. Apparently, none of the literature has suggested using the ideal pressure to perform these types of experiments. Several authors did not justify the use of higher pressure in their published study. It can be concluded that the use of different pressures in these studies is subjective rather than objective based. However, a higher pressure would tend to increase the permeability value of the dentine [20]. It would reduce the measuring time for each specimen allowing the researcher to use more samples to improve the power of the study [19].

The experimental protocol for the present study was designed in a way that the experimental agents were subjected to different treatments to simulate the oral environment. Therefore, these agents underwent assessment to decrease dentine permeability by its interactions with the simulated environment. This study not only helps to identify the immediate effect (10 mins after application) but also the late effects (7 days) of the desensitizing agents by constant blocking of the dentinal tubules. Evaluation was carried on up to a 7-day period because, in a clinical situation, the patient will be given a weekly review appointment to monitor the progress. Subsequently, these desensitizing agents were also challenged with citric acid for 3 minutes. This was to mimic the consumption of acidic beverages during daily life and record the interaction of desensitizing agents with it. Ideally, the best investigation to be carried out is *in vivo*, since it is difficult to set all the biological parameters to be in carried out *in vitro*. The researchers should be meticulous when deriving conclusions from these types of experimental outcomes since all these treatments just involved a single application of treatment agents and the methods did not evaluate any resistance of the treated dentine specimens to mechanical challenges.

The 2.7% wt. Mp-Mh oxalate showed the highest reduction in permeability compared to the other two products at all the stages of dentine permeability measurements. The permeability reduction values ranged between 92% and 81% (LpT3–LpT6). These results were in agreement with other studies reported in the literatures [21]. SEM images for dentinal tubule occlusion showed that calcium oxalate crystals did not occlude the tubules to a large extent and covered the significant dentine surfaces at all the stages of the dentinal treatments. As reported in a previous study, a good correlation could not be derived between the amount of crystals formed on the dentine surface and the percentage of reduction in dentine permeability for this solution [22]. However, the presence of oxalate deposits within the tubules in horizontal section would have narrowed the tubular diameter and reduced the permeability values. EDX analysis also showed the depletion of mineral content over time except for the immersion in artificial saliva for 10 minutes; this could be due to the transient incorporation of minerals from the artificial saliva. As reported in the literature, this solution has wide popularity because of its dual mechanism in treating dentine sensitivity by blocking excitation of the pulp sensory nerve and patent dentinal tubules [23]. To conclude from this investigation, the initial decrease in hypersensitivity after application is due to nerve depolarization by K^+^ ions and tubular occlusion, but the long-term effect of this product could be due to the deposition of calcium oxalate crystals within the tubules.

RMGI XT VAR showed significant reduction in the dentine permeability compared to LIQ SiO_2_ treatment. The percentage reduction of dentine permeability was ranged between 85% and 72% (LpT3–LpT6); this was similar to the value reported by a previous study [24]. SEM analysis showed the presence of cobblestone-like crystals covering the entire dentinal surface for the stages of treatment except for citric acid challenge. EDX analysis revealed the presence of its principal content such as Ca, F, Al, and Si in a high level at the application stage. However, during the subsequent stages, these minerals had started declining from its peak following the storage in artificial saliva for 10 minutes and 7 days. This could be due to the release of its minerals to the external environment after its interaction with artificial saliva. It could be assumed that the longer the immersion in an aqueous solution, the more the loss of mineral from the product, which could favor the saturation of tooth minerals during low pH within the mouth. Upon acid challenge, these materials further deteriorated from the superficial surfaces and eventual loss of minerals. However, SEM analysis showed some of the crystal plugs within the tubules to reduce the dentine permeability [25]. The horizontal section of the treated dentine specimen also showed some mineral crystals within the tubules. Appearance of resin tags within the tubules which was reported in a previous study could not be found in the present study [26]. Presumably, this would have been lost from the tubules when the specimen was sectioned. Furthermore, it has been reported that RM-GIC has reduced biocompatibility compared to conventional glass ionomer cements because of the presence of HEMA in their ingredients [27].

LIQ SiO_2_ showed lower reduction in permeability compared to the Mp-Mh oxalate and RMGI XT VAR. It reduced dentine permeability lower than 14% (LpT3 to LpT6). This material exceeded the maximum permeability value after citric acid challenge. SEM images show the precipitate of silica covering the dentine surface with a minimum number of tubules exposed for all the stages of dentine treatment except for the citric acid challenge. EDX analysis showed high peaks of Ca, P, and Si which were relatively constant throughout the dentine treatments. High peaks of minerals after immersion in artificial saliva depict that this material may have a remineralizing potential. However, the cross-section of the dentine specimen showed that the silica precipitate was not formed within the tubules. Overall, high permeability values could be explained due to the absence of the precipitate within the tubules that prevents the superficial silica deposits to have weak resistance to hydraulic conductance which leads to exposure of tubules and increased permeability. Another reason could be that the larger diameter silica deposits do not fit within the dentine tubules and eventually increase the fluid movements. To best of our knowledge, no studies have been published related to dentine permeability for this material. In addition, the biocompatibility of this agent is not reported in the literature.

### 4.1. Limitations

Since it is an *in vitro* experimental study, results should be interpreted meticulously. All the desensitizing agents were applied at one point. It may need several applications to improve its effectiveness. Additionally, it is difficult to determine the interaction of these agents with natural saliva in an *in vitro* test. Further well-controlled clinical trials are required to prove its clinical efficacy.

## 5. Conclusions

Within the limitation of the study, it could be concluded that 2.7% monopotassium-monohydrogen oxalate was significantly effective in reducing dentine permeability compared to RMGI XT VAR and LIQ SiO_2_.

## Figures and Tables

**Table 1:** Active ingredients and application procedures of the desensitizing agents used in the study.

Desensitizing agents	Manufacturer	Active ingredients	Application procedures
2.7% wt. monopotassium-monohydrogen oxalate pH 2.4 (Mp-Mh oxalate)	Experimental Solution	Potassium oxalate, monosubstituted hydrogen salts	Apply using the brush and air-dry

Clinpro™ XT Varnish (RMGI XT VAR)	3M ESPE, USA	Tube A: glass particles of silanized fluoroaluminosilicate, HEMA, water, BIS-GMA and silanized silica Tube B: copolymer of polyalkenoic acid, water, HEMA, and calcium glycerophosphate	Mix the pastes for 15 seconds, apply thin layer using brush, and light cure for 20 seconds

Dentcoat™ DSP (LIQ SiO_2_)	Andjana Medical Germany UG	Activator: alcohol, hydrochloric acid (pH 3) SiO_2_-complex: alcohol, aminoprophyl triethoxysilane	Mix the liquids, wait for 5–10 minutes, and apply using the applicator

**Table 2:** Dentine permeability (Lp) values expressed in percentage (%) after various treatment stages.

Treatments (LpT)	2.7% wt. Mp-Mh oxalate (group 1)(%)	RMGI XT VAR (group 2) (%)	LIQ SiO_2_ (group 3) (%)
Smear layer LpT1	23.35 ± 2.48	22.00 ± 1.77	21.50 ± 1.60
Acid etchant application LpT2	100 ± 0	100 ± 0	100 ± 0
Treatment application LpT3	8.25 ± 1.77^a^	14.89 ± 1.87^b^	85.24 ± 40.63^c^
Artificial saliva immersion—10 minutes LpT4	14.44 ± 1.45^a^	24.06 ± 2.29^b^	75.65 ± 38.88^c^
Artificial saliva immersion—7 days LpT5	13.09 ± 3.53^a^	22.35 ± 1.81^b^	97.83 ± 45.84^c^
Citric acid challenge—3 minutes LpT6	18.91 ± 3.34^a^	27.16 ± 1.09^b^	101.74 ± 43.94^c^

Values identified with different letters denote significant differences between and within the groups.

## Data Availability

The data used to support the findings of this study is confidential, and the availability of the data is restricted to the vicinity of University of Malaya. Hence, it cannot be made available upon request.
